# The proportions of people living with HIV in low and middle-income countries who test tuberculin skin test positive using either a 5 mm or a 10 mm cut-off: a systematic review

**DOI:** 10.1186/1471-2334-13-307

**Published:** 2013-07-08

**Authors:** Andrew D Kerkhoff, Ankur Gupta, Taraz Samandari, Stephen D Lawn

**Affiliations:** 1 School of Medicine and Health Sciences, The George Washington University, Washington, DC, USA; 2 Desmond Tutu HIV Centre, Institute of Infectious Disease and Molecular Medicine, Faculty of Health Sciences, University of Cape Town, Cape Town, South Africa; 3 Department of Clinical Research, Faculty of Infectious and Tropical Diseases, London School of Hygiene and Tropical Medicine, London, UK; 4 Division of Tuberculosis Elimination, Centers for Disease Control and Prevention, Atlanta, Georgia, USA

**Keywords:** HIV, Tuberculin skin test, TST, Tuberculosis, CD4

## Abstract

**Background:**

A positive tuberculin skin test (TST) is often defined by skin induration of ≥10 mm in people who are HIV-seronegative. However, to increase sensitivity for detection of *Mycobacterium tuberculosis* infection in the context of impaired immune function, a revised cut-off of ≥5 mm is used for people living with HIV infection. The incremental proportion of patients who are included by this revised definition and the association between this proportion and CD4+ cell count are unknown.

**Methods:**

The literature was systematically reviewed to determine the proportion of people living with HIV (PLWH) without evidence of active tuberculosis in low and middle-income countries who tested TST-positive using cut-offs of ≥5 mm and ≥10 mm of induration. The difference in the proportion testing TST-positive using the two cut-off sizes was calculated for all eligible studies and was stratified by geographical region and CD4+ cell count.

**Results:**

A total of 32 studies identified meeting criteria were identified, providing data on 10,971 PLWH from sub-Saharan Africa, Asia and the Americas. The median proportion of PLWH testing TST-positive using a cut-off of ≥5 mm was 26.8% (IQR, 19.8-46.1%; range, 2.5-81.0%). Using a cut-off of ≥10 mm, the median proportion of PLWH testing TST-positive was 19.6% (IQR, 13.7-36.8%; range 0–52.1%). The median difference in the proportion of PLWH testing TST-positive using the two cut-offs was 6.0% (IQR, 3.4-10.1%; range, 0–37.6%). Among those with CD4+ cell counts of <200, 200–499 and ≥500 cells/μL, the proportion of positive tests defined by the ≥5 mm cut-off that were between 5.0 and 9.9 mm in diameter was similar (12.5%, 12.9% and 10.5%, respectively).

**Conclusions:**

There is a small incremental yield in the proportion of PLWH who test TST-positive when using an induration cut-off size of ≥5 mm compared to ≥10 mm. This proportion was similar across the range of CD4+ cell strata, supporting the current standardization of this cut-off at all levels of immunodeficiency.

## Background

Tuberculin skin tests (TSTs) have for a long time been used to define likely *Mycobacterium tuberculosis (MTB)* infection, identify those who may benefit from isoniazid preventive therapy and aid diagnosis of tuberculosis (TB) disease, especially in children. While interferon gamma release assays (IGRAs) were developed to improve the diagnostic accuracy for MTB infection, the World Health Organization (WHO) does not recommend routine use of IGRAs in resource limited settings due to cost, technical complexity and limited utility [1].

The sensitivity of TSTs for detecting MTB infection is impaired among people living with HIV (PLWH) due to an attenuation of cell-mediated responses, including a reduction in memory CD4+ T cells at the TST site [2]. While a cut-off of ≥10 mm of induration is often used to define a positive TST among those who are HIV sero-negative, a cut-off of ≥5 mm is instead recommended by the US Centers for Disease Control and Prevention among PLWH to increase the sensitivity [3]. This revised cut-off is based on unpublished data from New York City in the 1980’s among just 23 AIDS patients with known TB disease of whom two had a TST of 5–9 mm induration while 13 had an induration ≥10 mm [4]. However, the incremental proportion of individuals defined as TST-positive when using this revised cut-off and the variables such as CD4+ cell count that might be associated with this proportion are not known.

Cobelens and colleagues [5] investigated TST responses among a large number of PLWH who were diagnosed as having active smear-positive pulmonary tuberculosis (TB) in Tanzania. Although just 71.2% of patients tested TST-positive when using the recommended cut-off of ≥5 mm of induration, this did not differ substantially from the proportion defined as positive using a cut-off of ≥10 mm of induration (64.3%) with a difference of just 6.9%. Thus, the altered cut-off only marginally reduced the number of false-negative tests. The authors concluded from their data that loss of sensitivity of the TST in PLWH is predominantly attributable to anergy (ie an all-or-nothing phenomenon) rather than due to a gradual diminution in TST diameter during progressive immunodeficiency. However, this study was conducted solely among PLWH who had active TB and these findings cannot necessarily be extrapolated to those with latent MTB infection and any association with CD4+ cell count was not defined.

We previously conducted a systematic review of the literature to ascertain the proportions of PLWH who tested TST-positive using the conventional cut-off of ≥5 mm of induration [6]. In the present systematic review, we determined the proportions of PLWH testing TST-positive using cut-offs of ≥5 mm and ≥10 mm cut-off and the relationship with CD4+ cell count.

## Methods

### Search strategy

The search strategy has been described in detail elsewhere [6]. In brief, we searched Embase, Global Health, Medline and Web of Science for relevant citations using a combination of MeSH and free text search terms according to a predetermined search criteria. The search was restricted to articles from January 1, 1990 to February 5, 2012 as an initial search demonstrated no relevant citations prior to this period. The first search set was created using comprehensive search terms for tuberculin skin test, latent tuberculosis and isoniazid preventive therapy and were combined using “or.” A second set was created for HIV/AIDS using comprehensive search terms. These two sets were then combined using “and.” Next, this set was combined with a low and middle-income country (LMIC) set (using the World Bank list of economies as of 01 July 2011) [7] using “and.” All citations were then limited to articles published in English and among humans.

Studies eligible for inclusion were those that were conducted in LMIC’s, included only PLWH who were at least 15 years of age, presented data for patients without evidence of active TB disease, and presented the proportion of those testing TST-positive current using a induration cut-off size of both ≥5 mm and ≥10 mm. Studies were also required to have at least 30 participants meeting the above criteria to limit imprecision around the point estimates. Studies utilizing two-step TST testing were excluded. All studies meeting the aforementioned criteria were included regardless of patient ART status at enrolment.

Citations identified through the search process were compiled into Endnote where duplicate citations were removed. Titles and abstracts were screened for study inclusion. Potentially relevant full-text citations were entered into an Excel spreadsheet and the full-texts were obtained. Two readers (ADK and SDL) carefully reviewed all full-text publications for study inclusion. Additional studies met the inclusion criteria following provision of additional data by the authors as previously described [6].

### Data extraction

Study characteristics from eligible studies were abstracted directly into a structured Microsoft Excel spreadsheet. Variables recorded included: title, authors, year of publication, study period, study location, clinical setting, mean or median patient age, bacille Calmette et Guerin (BCG) vaccine scar status, ART status, special or potentially non-generalizable populations and the total number of eligible PLWH who underwent TST testing (and returned to have their results read) and the total number PLWH testing TST-positive using both a ≥5 mm and a ≥10 mm induration size. When TST-results using both a ≥5 mm and a ≥10 mm cut-off stratified by CD4+ cell counts (<200, 200–499 and ≥500 cells/μL) were available, these were also recorded.

The quality of studies was independently assessed by two reviewers (ADK and SDL) using a graded checklist [6]. A study receiving 70% of available points was considered to be of “good quality”, 50-70% was considered “medium quality” and less than 50% was considered “lower quality.” We also assessed the risk of bias in individual studies as well as across all included studies.

### Statistical analysis

All statistical analysis was completed using STATA version 10.0. The difference in the proportion of those testing TST-positive using a ≥5 mm versus a ≥10 mm cut-off was calculated for all eligible studies and chi-squared tests were used to compare these proportions. Differences in the proportions of PLWH testing TST-positive by each cut-off size were then plotted using a forest plot and were stratified by geographic region and CD4+ cell count. Pooled estimates of overall data or stratified data were not derived due to substantial heterogeneity between studies as indicated by large I^2^ values [8].

## Results

**Figure 1:**
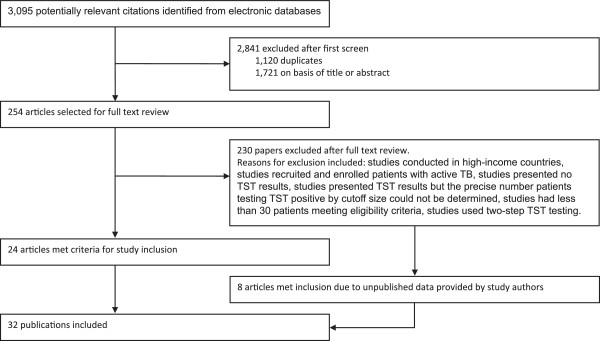
Overview of eligible studies containing data on the proportion of people living with HIV testing TST-positive using a ≥5 mm and ≥10 mm cut-off size.

Of the 3,095 potential citations identified in the literature search, 254 were selected for full-text review (Figure 1). After concluding a full-text review, 24 articles met the pre-defined inclusion criteria and an additional 8 articles met inclusion criteria due to unpublished data provided by authors for a previous study. In all, 32 publications provided data from 34 unique study populations and were published between 1992 and 2011 (Additional file 1). Among included publications, 14 study populations were from Africa, 8 from the Americas and 12 from Asia. The TB prevalence in counties in which the studies were conducted ranged from 0.19-785 cases per 100,000 population and the majority (62%; 21/34) of study populations were from high-prevalence TB countries (estimated TB prevalence >100 cases per 100,000 population).

The mean/median age among patients in eligible studies ranged from 22–42 years. The proportions of PLWH on ART at the time of TST assessment was reported for 18 study populations and an additional six study populations were assumed to be ART naïve as the studies were conducted prior to national ART implementation. Among these 24 populations, ART was received by a proportion of the participants in only 9 studies and this proportion varied from 1% to 100% (median 58%). Nine publications were of high quality, 12 studies of medium quality and 11 studies of low quality, as determined by a quality assessment checklist.

### Difference in patients testing TST-positive using a ≥5 mm versus ≥10 mm cut-off size

Overall, there were 10,971 PLWH with TST results from 34 study populations; 4,880 were from sub-Saharan Africa, 2,247 from the Americas and 3,844 from Asia. All studies reported the proportion of individuals testing TST-positive using both a 5 mm and 10 mm cut-off (Table 1).

**Table 1:** The proportion of people living with HIV who tested TST-positive using a ≥5 mm and ≥10 mm cutoff size and the difference between these two proportions

**Study**	**Number of patients (n)**	**Number of patients TST-positive using ≥5 mm cut-off (%)**	**Number of patients TST-positive using ≥10 mm cut-off (%)**	**Difference in proportion of patients testing TST-positive (%)**	**P-value**
**Africa**					
Allen et al. [9]	284	73 (25.7)	60 (21.1)	4.6	0.1977
Aisu et al. [10]	1094	579 (52.9)	376 (34.4)	18.6	<0.0001
Duncan et al. [11]	106	35 (33.0)	32 (30.2)	2.8	0.6576
Fordham von Reyn et al. [12]	104	21 (20.2)	20 (19.2)	1.0	0.8616
Diagbouga et al. [13]	37	9 (24.3)	6 (16.2)	1.7	0.3857
Waddell et al. [14]	58	16 (27.6)	15 (25.9)	8.1	0.8338
Mtei et al. [15]	460	192 (41.7)	175 (38.0)	3.7	0.2524
Tegbaru et al. [16]	116	47 (40.5)	38 (32.8)	7.8	0.2201
Rangaka et al. [17]	67	35 (52.2)	33 (49.3)	3.0	0.7297
Karam et al. [18]	273	59 (21.6)	46 (16.8)	4.8	0.1581
Hanifa et al. [19]	33	18 (54.5)	14 (42.4)	12.1	0.3245
Leidl et al. [20]	89	42 (47.2)	35 (39.3)	7.9	0.2896
Oni et al. [21]	240	138 (57.5)	125 (52.1)	5.4	0.2332
Samandari et al. [22]	1919	468 (24.4)	383 (20.0)	4.4	0.0010
**The Americas**					
Espinal et al. [23]	86	50 (58.1)	40 (46.5)	11.6	0.1269
Fordham von Reyn et al. [12]	121	3 (2.5)	2 (1.7)	0.8	0.6513
Garcia-Garcia et al. [24]	801	261 (32.6)	174 (21.7)	10.9	<0.0001
Miranda et al. [25]	98	16 (16.3)	12 (12.2)	4.6	0.4142
Balcells et al. [26]	109	12 (11.0)	7 (6.4)	4.1	0.2299
Gutierrez et al. [27]	104	20 (19.2)	13 (12.5)	6.7	0.184
Baboolal et al. [28]	64	6 (9.4)	0	9.4	0.0121
Moura et al. [29]	864	170 (19.7)	152 (17.6)	2.1	0.2661
**Asia**					
Suwanagool et al. [30]	399	323 (81.0)	173 (43.4)	37.6	<0.0001
Yanai et al. [31]	217	46 (21.2)	32 (14.7)	6.5	0.0801
Yanai et al. [31]	129	21 (16.3)	15 (11.6)	4.7	0.281
Sawhney et al. [32]	396	103 (26.0)	62 (15.7)	10.4	0.0003
Gupta et al. [33]	752	157 (20.9)	137 (18.2)	2.7	0.1935
Swaminathan et al. [34]	631	269 (42.6)	227 (36.0)	6.7	0.0155
Davarpanah et al. [35]	173	64 (37.0)	23 (13.3)	23.7	<0.0001
Davarpanah et al. [36]	459	131 (28.5)	60 (13.1)	15.5	<0.0001
Jiang et al. [37]	46	27 (58.7)	17 (37.0)	21.7	0.0369
Zhang et al. [38]	93	3 (3.2)	3 (3.2)	0	1
Kabeer et al. [39]	180	33 (18.3)	27 (15.0)	3.3	0.3961
Nguyen et al. [40]	369	204 (55.3)	175 (47.4)	8.1	0.0274

The median proportion of PLWH testing TST-positive using a cut-off of ≥5 mm was 26.8% (IQR, 19.8-46.1%; range 2.5-81.0%). There was considerable heterogeneity with regard to regional differences in the proportion of patients testing TST-positive. The median proportions of patients testing TST-positive in sub-Saharan Africa, the Americas and Asia were 36.8% (IQR, 24.7-51.0%; range, 20.2-57.5%), 17.8% (IQR, 10.6-22.9%; range, 2.5-58.1%), and 27.3% (IQR, 20.3-45.8%; range, 3.2-81.0%), respectively.

When a cut-off of ≥10 mm was used, the median proportion of patients testing TST-positive was 19.6% (IQR, 13.7-36.8%; range, 0–52.1%). Strong regional differences in the proportion of patients TST-positive were again noted. Patients in sub-Saharan Africa had a median TST-positive proportion of 31.5% (IQR, 20.3-39.0%; range, 16.2-52.1%) compared to 12.4% (IQR, 5.2-18.6%; range, 0–46.5%) in the Americas and 15.4% (IQR, 13.3-36.3%; range, 3.2-47.4%) in Asia.

**Figure 2:**
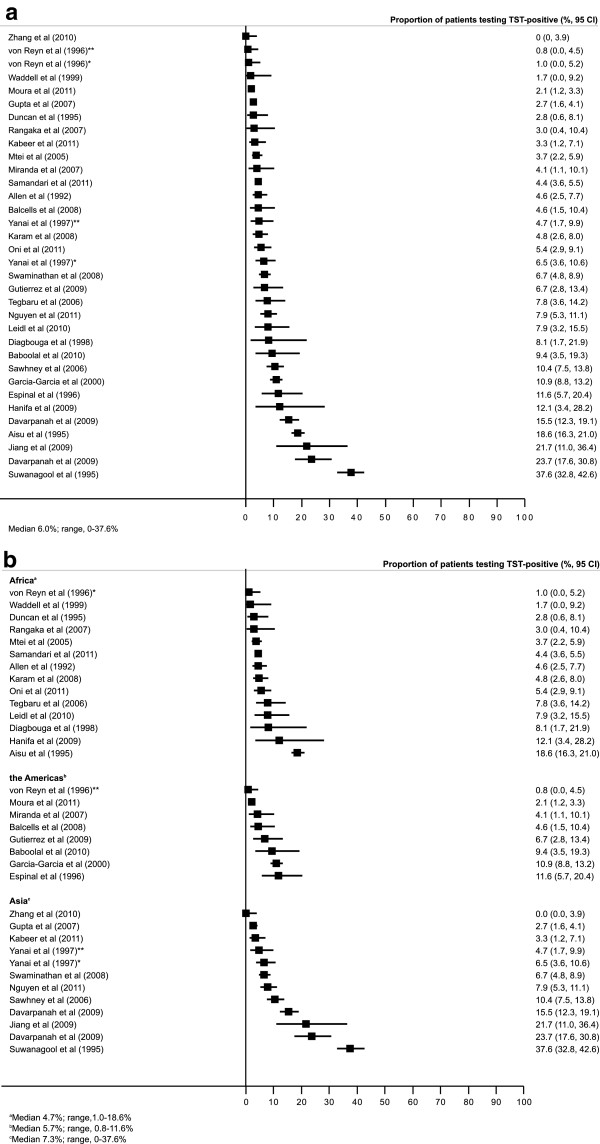
Forest plot showing the difference in proportions (%, 95% CI) of people living with HIV testing TST-positive using a ≥5 mm versus a ≥10 mm cut-off size in (a) all study populations (n = 34) and (b) with data grouped according to region.

The median difference observed in the proportion of those testing TST-positive using a cut-off size of ≥5 mm versus ≥10 mm was 6.0% (IQR, 3.4-10.1%; range 0–37.6%). When stratified by geographic region, the median difference in the proportion of patients testing TST-positive using a 5 mm versus a 10 mm cut-off was 4.7% (IQR, 3.1-7.9%, range, 1.0-18.6%), 5.7% (IQR, 3.6-9.8%, range, 0.8-11.6%) and 7.3% (IQR, 4.4-17.0%, range, 0–37.6%) for Africa, the Americas and Asia respectively. The overall difference in the proportions of patients testing TST positive (Figure 2a) and stratified according to geographic region (Figure 2b) was demonstrated in forest plots.

### Association with CD4+ cell counts

**Figure 3:**
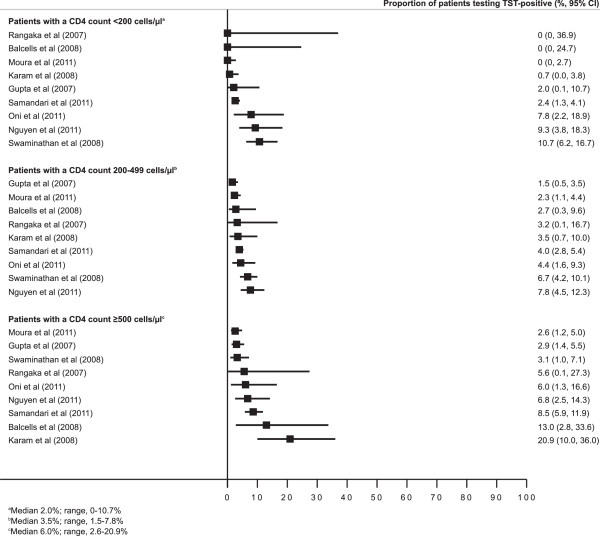
Forest plot showing the difference in proportions (%, 95% CI) of people living with HIV testing TST-positive using a ≥5 mm versus a ≥10 mm cut-off size with data grouped according to CD4+ cell count strata (<200, 200–499 and ≥500 cells/μL).

Nine studies with a total of 5,107 PLWH reported the proportion of those testing TST-positive using both a ≥5 mm and a ≥10 mm cut-off size stratified by CD4+ cell counts at <200, 200–499, ≥500 (Table 2). These CD4+ cell count stratified data are shown in a forest plot (Figure 3). The median difference in the proportion of PLWH testing TST-positive using the two induration cut-off sizes was 2.0% (range, 0–10.7%; IQR, 0–7.8%), 3.5% (range, 1.5-7.8%; IQR, 2.7-4.4%) and 6.0% (range, 2.6-20.9%; IQR, 4.9-9.6%) among those with CD4+ cell counts <200, 200–499, ≥500 cells/μL, respectively. The corresponding proportions of positive tests defined by the ≥5 mm cut-off that were between 5.0 and 9.9 mm in diameter were 12.5% (IQR, 0–16.0), 12.9% (IQR, 7.6-15.2), 10.5% (IQR, 9.1-28.5), respectively [9-40].

**Table 2:** The proportion of people living with HIV who tested TST-positive using a ≥5 mm and ≥10 mm cutoff size stratified by CD4+ cell count categories <200, ≥200, 200–499 and ≥500 cells/μL

**Study**	**Number of patients with CD4+ cell count <200**	**Number of patients TST positive using ≥5 mm cutoff (%)**	**Number of patients TST positive using ≥10 mm cutoff (%)**	**Number of patients with CD4+ cell count 200-499**	**Number of patients TST positive using ≥5 mm cutoff (%)**	**Number of patients TST positive using ≥10 mm cutoff (%)**	**Number of patients with CD4+ cell count ≥500**	**Number of patients TST positive using ≥5 mm cutoff (%)**	**Number of patients TST positive using ≥10 mm cutoff (%)**
Gupta et al. [33]	50	8 (16.0)	7 (14.0)	326	71 (21.8)	66 (20.2)	307	65 (21.2)	56 (18.2)
Rangaka et al. [17]	8	2 (25.0)	2 (25.0)	31	17 (54.8)	16 (51.6)	18	11 (61.1)	10 (55.6)
Karam et al. [18]	145	11 (7.6)	10 (6.9)	85	25 (29.4)	22 (25.9)	43	23 (53.5)	14 (32.6)
Balcells et al. [26]	13	1 (7.7)	1 (7.7)	73	4 (5.5)	2 (2.7)	23	7 (30.4)	4 (17.4)
Swaminathan et al. [34]	150	64 (42.7)	48 (32.0)	313	138 (44.1)	117 (37.4)	160	67 (41.9)	62 (38.8)
Moura et al. [29]	137	13 (9.5)	13 (9.5)	386	70 (18.1)	61 (15.8)	341	87 (25.5)	78 (22.9)
Nguyen et al. [40]	75	31 (41.3)	24 (32.0)	206	116 (56.3)	100 (48.5)	88	57 (64.8)	51 (58.0)
Oni et al. [21]	51	25 (49.0)	21 (41.2)	137	79 (57.7)	73 (53.3)	50	33 (66.0)	30 (60.0)
Samandari et al. [22]	575	88 (15.3)	74 (12.9)	952	250 (26.3)	212 (22.3)	364	124 (34.1)	93 (25.6)

## Discussion

This study found that among 10,971 patients without active TB in 34 different study populations, the incremental yield of patients defined as TST-positive when using a ≥5 mm cut-off rather than a ≥10 mm cut-off was just 6.0%. This suggests that using the modified cut-off had only a modest effect on increasing the proportion of PLWH who tested TST-positive. Importantly, this proportion was similar across the range of CD4+ cell count strata and also across different geographic regions.

Our findings of a 6% incremental proportion associated with the use of the revised TST cut-off among patients without active TB are very similar to those of Cobelens and colleagues [5] who reported a 7% increment among patients with active TB in Tanzania. This suggests that the small differences observed in the proportion of PLWH testing TST-positive using the two cut-off sizes are generalizable regardless of TB infection or disease status.

A recent systematic review and meta-analysis [41] investigated the utility of ≥5 mm and ≥10 mm TST cut-off sizes to predict the progression from latent infection to active TB disease. Among five studies in which the HIV status of patients was largely unknown, the unadjusted incidence rate ratio of tuberculosis in TST-positive patients was 1.43 (95% CI, 0.75-2.72) when a cut-off of ≥5 mm was used and 1.60 (95% CI, 0.94-2.72) when a cut-off ≥10 mm was applied. Thus, neither cut-off size better predicted the risk for progression to active TB disease. However, because the HIV status of the patients included in these studies was negative or largely unknown, it remains unclear whether these findings are generalizable to PLWH.

TSTs rather than IGRAs are the WHO-recommended assay for use in LMICs for detecting latent tuberculosis infection among PLWH prior to isoniazid preventive therapy [42]. Although use of TST is recommended, it remains unknown whether a ≥5 mm or ≥10 mm cut-off size better defines those who will benefit from IPT. A meta-analysis [43] among PLWH demonstrated that among those with a TST induration of ≥5 mm in diameter, IPT was associated with a TB risk reduction of 64% (95% CI, 39-78 %). However, all studies in this meta-analysis defined TST-positivity using a ≥5 mm cut-off size and thus it is not known whether those with a 5.0-9.9 mm induration gain benefit from IPT. An individual patient meta-analysis of data from randomized trials of isoniazid preventive therapy may be able to define this.

Strengths of this study include a comprehensive search strategy that utilized four different study databases. There were more than 10,000 patients from 34 unique study populations from many countries and geographic regions. Also, a number of variables potentially influencing differences in the proportion of TST-positive patients were recorded and summarized where possible. There were also several limitations. The reliability of TST assessment could not be assessed. Additionally, methods for excluding TB disease were not standardized among included studies. Heterogeneity in the data precluded the calculation of pooled summary estimates.

## Conclusion

In conclusion, using a ≥5 mm cut-off among PLWH resulted in only a modest increase in the proportion testing TST-positive when compared to a cut-off size of ≥10 mm and this finding concurs with a similar study that was conducted among patients with active TB. The difference in the proportion of PLWH testing TST-positive when using the two different cut-off sizes was similar across the range of CD4+ cell count strata suggesting use of this cut-off across the spectrum of immunodeficiency.

## Competing interest

The authors have no conflicts of interest to declare.

## Authors’ contributions

ADK initiated the study. ADK designed the search strategy. ADK ran the searches and selected and graded the studies with SDL. ADK, AG and SDL did the data synthesis. ADK and SDL wrote the manuscript and all other co-authors contributed to and approved the final draft.

## Pre-publication history

The pre-publication history for this paper can be accessed here:

http://www.biomedcentral.com/1471-2334/13/307/prepub

## Supplementary Material

Additional file 1 Characteristics of studies presenting data on the proportion of HIV-infected patients who tested TST-positive using a ≥5 mm and ≥10 mm cut-off size. Click here for file
