# Supplementary material for: The proportions of people living with HIV in low and middle-income countries who test tuberculin skin test positive using either a 5 mm or a 10 mm cut-off: a systematic review
Source: BMC Infect Dis. 2013 Jul 8;13:307. doi: 10.1186/1471-2334-13-307 (PMC3716635; doi:10.1186/1471-2334-13-307)
Supplement: Additional file 1 — Characteristics of studies presenting data on the proportion of HIV-infected patients who tested TST-positive using a ≥5 mm and ≥10 mm cut-off size. [file 1471-2334-13-307-S1.doc]

**Additional file 1: Characteristics of studies presenting data on the proportion of HIV-infected patients who tested TST-positive using a ≥5 mm and ≥10 mm cut-off size**

| **Study** | **Country** | **Study years** | **Five year average TB prevalence rate (per 100,000)** | **Study setting** | **Eligible patients (N)** | **Mean age (years)** | **Study population** | **Special populations** | **Methods used to exclude active TB among study patients** | **TST methods** | **Patients on ART at enrolment (%)** | **Patients with at least one BCG scar (%)** | **TST results stratified by CD4+ count available?** | **Secondary data provided for study inclusion?** |
| --- | --- | --- | --- | --- | --- | --- | --- | --- | --- | --- | --- | --- | --- | --- |
| **Africa** | | | | | | | | | | | | | | |
| Allen et al (1992)[9] | Rwanda (Kigali) | 1986-1987 | 14 | Urban | 284 | .. | Outpatient paediatric and prenatal clinics at community hopsital | Only women were included | Symptom screen, clinical investigation, chest X-ray, sputum smear and culture | 1-TU PPD RT23 read after 48-72 hours | pre-ART era | 78 | No | No |
| Aisu et al (1995)[10] | Uganda (Kampala and Nakulabye) | 1991-1992 | 104 | Urban | 1094 | .. | HIV testing center | Only patients age 15-45 were included | Symptom screen, chest X-ray and sputum smear | 2-TU PPD RT23 in Tween 80 read after 48-72 hours | pre-ART era | 61 (among TST-positive only) | No | No |
| Duncan et al (1995)[11] | Zambia (Lusaka) | 1990 | 23 | Urban | 106 | .. | STD clinic within a university hospital | … | … | 0.1 mL 2-TU PPD RT23 read after 48 hours | pre-ART era | … | No | No |
| Fordham von Reyn et al (1996)[12] | Kenya (Nairobi) | 1991-1994 | 66 | Urban | 104 | 34 | Outpatient clinic and inpatient services of hospital | Only patients with CD4+ count <200 were included | … | 0.1 mL 5-TU Tubersol PPD (Connaught Laboratories) read after 48–72 hours | 0 | 39 | No | No |
| Diagbouga et al (1998)[13] | Burkino Faso (Bobo-Dioulassa) | 1994-1995 | 19 | Urban | 37 | 34.5 | STD/AIDS clinic | … | Clinical investigation | 10-TU tuberculin (Pasteur Mérieux Serums et Vaccins) read after 72 hours | pre-ART era | … | No | No |
| Waddell et al (1999)[14] | Zambia (Lusaka) | … | 66 | Urban | 58 | 34.6 | Individuals attending one of three settings offering HIV services | Only patients with CD4+ count ≥200 were included | Two sputum smears | 0.1 mL 5-TU Tubersol PPD (Connaught Laboratories) read after 48–72 hours | … | 38 | No | No |
| Mtei et al (2005)[15] | Tanzania  (Dar es Salaam) | 2001-2003 | 239.0 | Urban | 460 | 34* | Recently diagnosed patients referred from HIV testing centers | Only patients with CD4+ count >200 were included | Symptom screen, clinical examination, chest X-ray, sputum smear and culture | PPD RT23 | 0 | 100 | No | No |
| Tegbaru et al (2006)[16] | Ethiopia (Addis Ababa) | 1995-1997 | 375.0 | Urban | 116 | 33 | Factory workers who were living in and around Addis Ababa | Only factory workers were included | Clinical examination, chest X-ray, sputum smear and culture | 0.1mL 2-TU PPD RT23 | 0 | … | No | No |
| Rangaka et al (2007)[17] | South Africa (Khayelitsha) | … | 654.5 | Urban | 67 | 30 | Individuals attending an integrated HIV-TB clinic for HIV testing | … | Symptom screen and clinical examination or Karnofsky score <60 | 2-TU PPD RT23 | 0 | 51 | Yes | No |
| Karam et al (2008)[18] | Senegal (Dakar) | 2003-2005 | 462.0 | Urban | 273 | 37* | Individuals attending an infectious disease department or ambulatory care centre of a hospital | … | Clinical, radiological or microbiological evidence or Karnofsky score <80 | 2-TU PPD RT23 read after 48–72 hours | 0 | 73 | Yes | Yes |
| Hanifa et al (2009)[19] | South Africa | 2005-2006 | 654.5 | … | 33 | … | Employees from gold mines | Only miners were included | … | 2-TU PPD RT23 in Tween 80 read after 48-72 hours | … | … | No | No |
| Leidl et al (2010)[20] | Uganda (Kampala) | … | 313.8 | Urban | 89 | 34.1 | HIV outpatient clinic | … | Symptom screen, clinical examination, chest X-ray, sputum examinations, or Karnofsky score <60 | 0.1 mL 2-TU PPD RT23 read after 48 hours | 0 | … | No | No |
| Oni et al (2011)[21] | South Africa (Khayelitsha) | 2008-2010 | 785.0 | Urban | 238 | 30* | Individuals attending an integrated HIV-TB clinic pre ART initiation | … | Symptom screen, chest X-ray (if TST+) and sputum smear and culture | 2-TU PPD RT23 read after 48–72 hours | 0 | 54 | Yes | Yes |
| Samandari et al (2011)[22] | Botswana (Gaborone and Francistown) | 2004-2009 | 750.0 | Urban | 1919 | 34 | Government clinics providing ART and IPT services | … | Symptom screen and chest X-ray | 5-TU PPD RT23 read after 48–72 hours | 2 | 78 | Yes | Yes |
| **The Americas** | | | | | | | | | | | | | | |
| Espinal et al (1996)[23] | Dominican Republic (Santo Domingo) | 1992-1994 | 23 | Urban | 86 | 29.2 | HIV testing center | Only women were included | Clinical investigation, chest X-ray and three sputum smears | 5-TU Applisol PPD (Parke-Davis) | pre-ART era | … | No | No |
| Fordham von Reyn et al (1996)[12] | Trinidad (Port of Spain) | 1992-1994 | 0.19 | Urban | 121 | 35 | Outpatient clinic and inpatient services of hospital | Only patients with CD4+ count <200 were included and 24% were MSM | … | 0.1 mL 5-TU Tubersol PPD (Connaught Laboratories) read after 48–72 hours | 1 | 44 | No | No |
| Garcia-Garcia et al (2000)[24] | Mexico (Mexico City) | 1992-1993 | 123 | Urban | 801 | 31 | HIV testing centre | 72.9% of patients were MSM and 1.1% were IDU | Clinical screen, chest X-ray, sputum smear and culture | 5-TU PPD RT23 read after 48–72 hours | 12 | 82 | No | No |
| Miranda et al (2007)[25] | Brazil (7 states) | 1995-2001 | 104 | … | 98 | 34* | Individuals attending a public HIV treatment facility | 59% of patients were MSM, 13% had a history IDU and 7% had a history of incarceration | Chest X-ray, sputum microscopy and sputum culture | … | 84 | … | No | Yes |
| Balcells et al (2008)[26] | Chile (Santiago) | 2006-2007 | 16.5 | Urban | 109 | 38.8* | HIV outpatient clinics | Only patients with CD4+ count >100 were included | Symptom screen | 2-TU PPD RT23 read after 48–72 hours | 58 | 84 | Yes | Yes |
| Gutierrez et al (2009)[27] | Brazil  (Sao Paulo) | 2005 | 75.5 | Urban | 104 | 41.6 | HIV/AIDS outpatient clinic | 56% of men were MSM and 5% of all patients were IDU | Methods unclear | … | 92 | … | No | No |
| Baboolal et al (2010)[28] | Trinidad and Tobarango | 2007 | 0.26 | … | 64 | … | Outpatient respiratory care clinics and a tertiary TB health center | … | Methods unclear | 0.1 mL Tubersol PPD (Connaught Laboratories) read after 72 hours | 100 | … | No | No |
| Moura et al (2011)[29] | Brazil (Recife) | 2007-2010 | 59.0 | Urban | 864 | 40.4 | Individuals in outpatient hospital services that serve as HIV/AIDS referral services | … | … | 0.1mL PPD RT23 read after 72 hours | 78 | … | Yes | Yes |
| **Asia** | | | | | | | | | | | | | | |
| Suwanagool et al (1995)[30] | Thailand (Banglok) | 1993-1994 | 208 | Urban | 399 | 24.2 | Individuals attending an infectious disease and counseling clinic | Only asymptomatic patients with a CD4+ count >200 were included | Chest X-ray | 10-TU PPD-TRC (Thai Red Cross) read after 48-72 hours | pre-ART era | 61 | No | No |
| Yanai et al (1997)[31] | Thailand Chiang Rai) | 1992-1994 | 208 | Urban | 217 | 28.7 | Individuals attending a blood bank | Only those attending a blood bank were included | … | 5-TU Tubersol PPD (Connaught Laboratories) read after 48–72 hours, but up to 5 days | … | 72 | No | No |
| Yanai et al (1997) [31] | Thailand Chiang Rai) | 1992-1994 | 208 | Urban | 129 | 22.3 | Female sex workers attending STD clinic | Only female sex workers were included | … | 5-TU Tubersol PPD (Connaught Laboratories) read after 48–72 hours, but up to 5 days | … | 62 | No | No |
| Sawhney et al (2006)[32] | India (Pune) | 1997-2002 | 241 | Urban | 396 | 33.8 | Individuals attending an armed services hospital | Only serving armed forces members were included and were 99.6% male | … | … | … | … | No | No |
| Gupta et al (2007)[33] | India (Pune) | 2002-2005 | 248 | Urban | 752 | 23* | Pregnant women attending a public hospital | Only pregnant women were included | Symptom screen, chest x-ray, sputum smear and culture | 5-TU PPD and read after 48–72 hours | … | … | Yes | Yes |
| Swaminathan et al (2008)[34] | India (Chennai) | 2000-2005 | 248 | Urban | 631 | 30 | Individuals attending government-funded TB clinics | … | Symptom screen, chest X-ray and 3 sputum cultures | 1-TU PPD RT23 and read after 48–72 hours | … | 48 | Yes | No |
| Davarpanah et al (2009)[35] | Iran (Shiraz) | 2008 | 36 | Urban | 173 | 38* | Individuals attending an infectious disease clinic | … | … | 5-TU PPD and read after 48–72 hours | … | … | No | No |
| Davarpanah et al (2009)[36] | Iran (Shiraz) | 2004-2006 | 47 | Urban | 459 | … | Counselling and behavioural modication center | Many patients were MSM or had a history of IDU | … | … | … | … | No | No |
| Jiang et al (2009)[37] | China (Yunnan Province) | 2002-2005 | 146.7 | … | 46 | 33.9 and 33.7 | Individuals from within a province of high drug abuse | … | Chest X-ray, sputum microscopy and sputum culture | 5-TU PPD RT23 read after 48–72 hours | **…** | … | No | No |
| Zhang et al (2010)[38] | China (Shenzhen) | … | 144.8 | Urban | 93 | 36 | Individuals attending an ART clinic | … | Symptom screen, clinical examination and chest X-ray | 5-TU PPD (Chengdu Institute of Biological Products) read after 72 hours | 0 | 100 | No | No |
| Kabeer et al (2011)[39] | India  (Chennai) | 2007-2009 | 252.5 | Urban | 180 | 36* | Government hospital of thoracic medicine | … | Clinical, radiological, and microbiological investigations | 2-TU PPD RT23 read after 48-72 hours | 0 | … | No | No |
| Nguyen et al (2011)[40] | Viet Nam (Ho Chi Minh City) | 2009-2010 | 334.4 | Urban | 369 | 30* | Individuals attending a public clinic that offers HIV services | 62.9% of patients were IDU, 15.5% had a history of incarceration, 44.2% have a history of TB | Symptom screen, chest X-ray, sputum smear and culture | 5-TU PPD read after 48–72 hours | 58.3 | 55.6 | Yes | Yes |

*Denotes median age.

“…” denotes information not stated.

*TU* tuberculin units, *PPD* purified protein derivative, *STD* sexually transmitted disease; *MSM* men who have sex with men;

*IDU* people who inject drugs, *ART* antiretroviral therapy, *IPT* isoniazid preventive therapy.
